# Psychological Distress Among Domestic Burglary Victims: A Systematic Review of Possible Risk and Protective Factors

**DOI:** 10.1177/15248380231155525

**Published:** 2023-02-27

**Authors:** Maarten Kunst, Dante Hoek

**Affiliations:** 1 Leiden University, Leiden, The Netherlands

**Keywords:** domestic burglary, risk factors, protective factors, psychological distress, review

## Abstract

Domestic burglary victimization is a potentially traumatic experience, because most people consider their home as an extension of the self and a place where the self is protected against others. Intrusions to such a highly valued place are therefore considered as attacks to both one’s personhood and one’s safety and privacy and may render victims at risk of psychological distress. In view of the legal obligations most countries have with regard to screening crime victims for psychological distress, this study systematically reviewed the literature on determinants of psychological distress in domestic burglary victims. Web of Science, EBSCO, and ProQuest databases and reference lists were searched between February and July 2022 to identify relevant studies. In all, 10 studies met all inclusion criteria and were evaluated according to the Cambridge Quality Checklists. These checklists have been developed to assess the methodological qualities of observational research. Findings of included studies suggest that female sex, damages caused by the burglary, and evaluations of the police response are potential determinants of psychological distress. However, given the dearth of research and the old age and theoretical and methodological limitations of included studies, it is too early to draw definite conclusions about the predictive value of these and other factors and to provide directions for screening policies. Future research should use prospective designs to overcome these limitations and ensure that domestic burglary victims at risk of psychological distress are timely referred to adequate professional help services.

## Introduction

Crime may have severe consequences for its victims; a large body of research has shown that many victims temporarily suffer from psychological distress and that some of them develop trauma-related disorders or mood or anxiety disorders, which may pose them at risk of social, financial, economic, and physical health problems (Hanson et al, 2010; Macmillan, 2000). In addition, numerous studies have revealed which victims run an increased or decreased risk of experiencing psychological distress. Knowledge about risk and protective factors is important to help law enforcement and victim support agencies to establish efficient and effective screening and referral strategies (Hanson & Self-Brown, 2010). Indeed, in many countries, these agencies need this knowledge to fulfill their legal obligations toward victims. After all, both the 1985 United Nations (UN) *Declaration of Basic Principles of Justice for Victims of Crime and Abuse of Power* (United Nations General Assembly, 1985) and the 2012 European Union *Directive establishing minimum standards on the rights, support and protection of victims of crime* (European Parliament and Council, 2012) require their member states to legally ensure that crime victims suffering from psychological distress are referred to and helped by professional support services (see Groenhuijsen, 2014).

For severe violent and sexual crimes, such as sexual assault against women, intimate partner violence, and child abuse, a number of systematic reviews have summarized and synthesized prior research to provide policymakers and practitioners with insight into possible determinants of psychological distress (e.g., Campbell et al., 2009; Yule et al., 2019). However, for other potentially impactful types of crimes, such reviews appear to be nonexistent. This study aims to start filling this gap in the literature by systematically reviewing research on possible risk and protective factors for psychological distress among victims of domestic burglary.

Domestic burglary is a huge societal problem in almost any country around the globe. Although many countries have experienced sharp decreases in burglary numbers in recent decades, in many places it is still among the most frequently occurring crimes. In the European Union around 1.5 million domestic burglaries are reported to the police each year and in the United States around 1.1 million (Eurostat, 2022; United States Department of Justice/Federal Bureau of Investigation, n.d.). The monetary costs of these burglaries (e.g., due to damaged properties and stolen goods, police investigations, victim services) are enormous. For example, the total monetary costs of domestic burglary have been estimated to amount to more than 9 billion dollars a year in the United States (which comes down to 2,675 dollars per burglarized house; see Miller et al., 2021) and slightly less than 2.4 billion pounds sterling for the United Kingdom (which comes down to 3,420 pounds sterling per burglarized house; see Heeks et al., 2018).

However, it is not just the monetary costs that makes domestic burglary a devastating experience for its victims. Prior research suggests that domestic burglary victimization may, at least for some victims, be just as psychologically damaging as the crimes mentioned above. Below we will discuss this research in more detail, but first we will present a theoretical framework that can help to understand the psychological impact of domestic burglary victimization.

### A Theory on the Psychological Impact of Domestic Burglary Victimization

The psychological impact of domestic burglary victimization can be explained by Ronnie Janoff-Bulman’s (1992) *Shattered Assumptions Theory*. According to this theory, distressing events cause a shattering of one’s fundamental assumptions about the world, others, and the self. This applies even more to criminal events, as such events typically target the person of the victim or his or her belongings (Janoff-Bulman, 1985). Brown and Harris (1989) have integrated Janoff-Bulman’s work with Altman’s concept of “territoriality”. Altman (1975) argued that many people see their home as a “primary territory”. In contrast to “secondary territories”, such as neighborhood streets, workplaces, and bars, and “tertiary territories”, such as public benches, primary territories are seen as an extension of the self and a place where the self is protected against others. Intrusions to such highly valued territories are potentially traumatic, because they shatter victims’ beliefs in the inviolability of these territories (e.g., Brown & Harris, 1989; Korosec-Serfaty & Bolitt, 1986). People who experience intrusions are thus at risk of posttraumatic stress disorder (PTSD) and other forms of psychological distress (Basdeo, 2020).

### Research on Domestic Burglary Victimization and Psychological Distress

Studies investigating psychological distress in the context of domestic burglary victimization can be divided into three categories: (1) crime survey studies, (2) in-depth interview studies, and (3) mental health survey studies. Studies belonging to the first category typically ask their respondents whether they have experienced a burglary within a specific time frame—usually the previous year—and, if so, then ask them to indicate whether and how the burglary has affected them. Based on these studies, we know that many domestic burglary victims experience feelings of anger, annoyance, fear, and depression in response to the burglary, and that some suffer from PTSD, anxiety, or panic attacks (e.g., Walker et al., 2006; Wohlfarth et al., 2003). Studies belonging to the second category typically interview (a small number of) domestic burglary victims to get more insight into these outcomes. In line with Brown and Harris’s (1989) integration of Janoff-Bulman’s shattered assumptions theory and Altman’s (1975) concept of territoriality, these studies have shown that for many victims domestic burglary is a traumatic experience, because it involves a violation of the safety and privacy of their home (e.g., Wollinger, 2015, 2017). Finally, studies belonging to the third category of studies are quantitative in nature and are mainly interested in domestic burglary victimization as a potential source of psychological distress. These studies can be further divided into studies which compare domestic burglary victims with victims of other types of crime on indices of psychological distress and studies which investigate domestic burglary victimization as a correlate or risk factor of psychological distress indices. Studies of the first subcategory suggest that, on average, domestic burglary victims experience less psychological distress than victims of violent and sexual crimes, but that some of them experience just as much psychological distress (e.g., Kunst & Koster, 2017; Lurigio, 1987). Studies of the second subcategory suggest that domestic burglary victimization is associated with a wide array of psychological distress indices, including fear of crime (e.g., Doyle et al., 2021), unhappiness (Staubli et al., 2014), dissatisfaction with life (e.g., Cohen, 2008), and anxiety and depression (e.g., Kilian et al., 2021). In some cases, these psychological costs may eventually result in social problems, such as divorce (e.g., Tark et al., 2008), behavioral problems, such as withdrawal and aggression (e.g., Ramey & Harrington, 2019), or physical ailments, such as cardiovascular health problems (e.g., Browning et al., 2012) and adiposity (e.g., Lee et al., 2019).

### The Current Study

To mitigate crime victims’ psychological distress, it is important that they receive adequate support services. To ensure that they have access to such services, police officers and other professionals working with crime victims should screen them for factors shown to be related to the experience or development of psychological distress (Winkel et al., 2003). As mentioned above, for severe and sexual crimes, it is known on which factors victims should be screened to enable referral to support services, but not for domestic burglary. The goal of this study is to fill this gap in the literature by systematically reviewing the literature on possible determinants of and protectors against psychological distress in domestic burglary victims.

## Methods

The review was performed in line with the Preferred Reporting Items for Systematic Review and Meta-Analysis Protocols (Moher et al., 2015).^1^

### Literature Search

To detect relevant studies, the following English language databases were searched between February and July 2022: Web of Science (all collections), EBSCO (including Academic Search Premier, APA PsycArticles, APA PsycInfo, Criminal Justice Abstracts, MEDLINE, Psychology and Behavioral Sciences Collection), and ProQuest (including Periodicals Archive Online, ProQuest Dissertations & Theses Global, PTSDpubs, Social Services Abstracts and Sociological Abstracts). The last search was performed on July 20th 2022.

In all databases, title, abstract, and subject fields were searched for combinations of the terms “burglary,” “risk factor,” “protective factor,” and “psychological distress” and synonyms of these terms.^2^

Titles and abstracts were screened to determine selection for full-text reading. To identify relevant studies which were not obtained through the search process, reference lists of included studies were carefully screened. Finally, Google Scholar was consulted to check publications which cited selected studies. After completion of the search process, full texts of potentially relevant publications were read to decide upon eligibility for inclusion. If full texts were not available from (university) libraries in the Netherlands, authors were asked to send an electronic or a hard copy print of the document of interest.

### Inclusion and Exclusion Criteria

Studies were included in the review if they (1) were written in English, (2) investigated the association between a potential risk or protective factor and an index of psychological distress among victims of domestic burglary, and (3) statistically tested for the significance of this association or provided enough data to calculate the significance of this association. Both retrospective and prospective studies were eligible for inclusion. Studies were excluded if results did not distinguish between victims of domestic burglary and victims of other types of crime. “Psychological distress” was defined as “the unique discomforting, emotional state experienced by an individual in response to a specific stressor or demand that results in harm, either temporary or permanent, to the person” (Ridner, 2004, p. 539).

### Methodological quality Assessment of Selected Studies

The Cambridge Quality Checklists were used to assess the methodological qualities of selected studies. These checklists have been developed to critically evaluate studies investigating associations between non-manipulated risk or protective factors and outcomes of interest (Murray et al., 2009) and have a high inter-rater reliability (Jolliffe et al., 2012).

#### Checklist for correlates

The checklist for correlates includes five items regarding sampling methods (adequate = total population sampling or random sampling [rated as 1], inadequate = convenience sampling or case control sampling [rated as 0]), response rates (adequate = response and retention rates ≥ 70% and differential attrition ≤ 10% [rated as 1], inadequate = response rate < 70%, or retention rate < 70, or differential attrition > 10% [rated as 0]), sample size (adequate = sample size ≥ 400 [rated as 1], inadequate = sample size < 400 [rated as 0]), and measurement of the correlate and the outcome (reliability coefficient ≥ .75 *and* reasonable face validity, or criterion or convergent validity coefficient ≥ .3, or more than one instrument or information source used to assess correlate/outcome [rated as 1], inadequate = none of the aforementioned [rated as 0]).

#### Checklist for risk/protective factors

The checklist for risk factors includes one item regarding the time-ordering of data and uses three response categories (rated as 1 = cross-sectional data, 2 = retrospective data, and 3 = prospective data). It allows for making a distinction between true risk or protective factors (i.e., factors which have been shown to precede the outcome of interest in time) and correlates (i.e., factors which have been shown to correlate with the outcome of interest in one and the same assessment).

#### Checklist for causal risk/protective factors

The checklist for causal risk factors includes one item regarding study design (rated as 1 = study with no comparison group—no analysis of change, 2 = inadequately controlled study—no analysis of change, 3 = uncontrolled study—with analysis of change, 4 = inadequately controlled study—with analysis of change, 5 = controlled non-experimental study—no analysis of change, 6 = controlled non-experimental study—with analysis of change, and 7 = randomized experiment targeting a risk factor; see Murray et al., 2009).^3^

### Data Analysis

All potentially relevant studies were independently assessed for eligibility by both authors. Disagreements were solved during a consensus meeting. After consensus was reached upon the studies to be included in the review, each author summarized the studies’ main results according to a predetermined format. Finally, a second consensus meeting was held to reach agreement on the results and quality of included studies.

Included studies were not subjected to a meta-analysis, because, as will become clear from the results section, there was a large heterogeneity between studies in terms of measurement and statistical methods. For example, most studies used different indices to measure psychological distress, used different instruments to measure the same risk or protective factor or the same index of psychological distress, and either confined statistical methods to bivariate analyses or also used more sophisticated analyses to test for significance of associations. Under these circumstances, a meta-analysis would yield unreliable results (cf. Hernandez et al., 2020).

## Results

**Figure 1.**
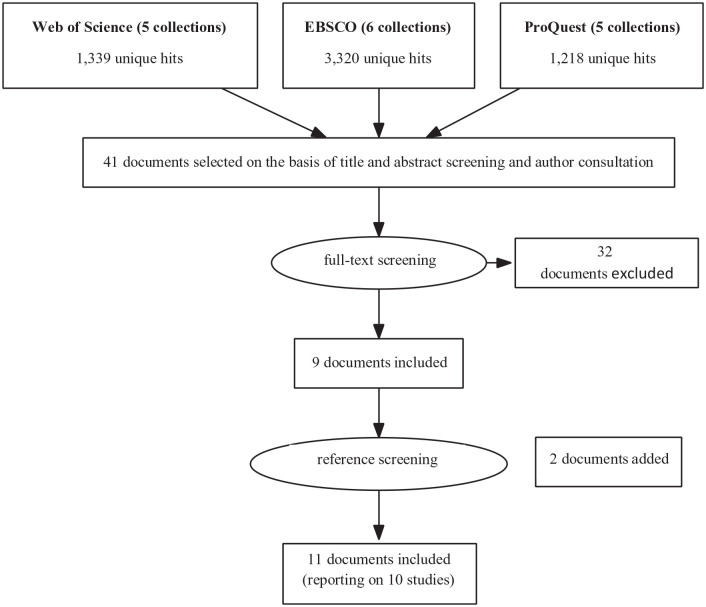
Flowchart of the literature search process.

The literature search yielded 5,877 hits. After removal of duplicates and initial screening on the basis of titles and abstracts, 41 documents were selected for full-text reading. One of these documents could not be obtained from a Dutch university library and was therefore requested from the authors (Kobayashi & Saito, 1995). The first author of this document let us know that it was written in Japanese and instead sent us an unpublished conference paper, which was written in English and based on the same data as the requested publication (Kobayashi, 1996). This unpublished conference paper and 10 journal articles were eventually included in the review. These 11 documents described 10 unique studies (see Figure 1).

Three studies were conducted in the United Kingdom (Beaton et al., 2000; Chung et al., 2014; Maguire, 1980), two in the Netherlands (Kunst et al., 2013; Winkel & Vrij, 1993; Winkel et al., 1994), one in the United States (Brown & Harris, 1989), one in Japan (Kobayashi, 1996), one in Canada (Waller & Okihiro, 1978), one in New Zealand (Wylie, 1993), and one in three different countries (Mawby et al., 1999). The number of participants ranged from 20 to 322. In total, 1,511 victims of domestic burglary participated in the studies. Remarkable was that all studies were rather old: the most recent study was conducted in 2014 and the eldest in 1978.

Since we deemed meta-analysis inappropriate, findings of included studies are summarized and synthesized in a narrative way (cf. Petticrew & Roberts, 2006). To structure the presentation of the results, we make a distinction between three categories of factors that may be associated with experiencing psychological distress: pre-burglary, peri-burglary, and post-burglary factors (cf. Denkers, 1996; Sales et al., 1984). But first we will briefly describe the studies’ methodological qualities.

### Methodological Qualities of Selected Studies

Four studies lected data through questionnaires (Beaton et al., 2000; Chung et al., 2014; Kobayashi, 1996; Winkel & Vrij, 1993 and Winkel et al., 1994), two through telephone interviews (Brown & Harris, 1989; Kunst et al., 2013), and four through face-to-face interviews (Maguire, 1980; Mawby et al., 1999; Waller & Okihiro, 1978; Wylie, 1993). As shown in Table 1, seven studies measured psychological distress with self-developed instruments (Brown & Harris, 1989; Kobayashi, 1996; Maguire, 1980; Mawby et al., 1999; Waller & Okihiro, 1978; Winkel & Vrij, 1993; Winkel et al., 1994; Wylie, 1993) and three with validated instruments (Beaton et al., 2000; Chung et al., 2014; Kunst et al., 2013).

**Table 1.** Summaries of Included Studies.

Study	Risk/Protective Factors	Psychological Distress
1. Beaton et al. (2000)	1. Female sex	1. Mental health problems as measured by the 12-item version of the GHQ (Goldberg, 1978)
		2. Mood states as measured by the six subscales (Composed–Anxious, Agreeable–Hostile, Elated–Depressed, Confident–Unsure, Energetic–Tired, and Clear-headed–Confused) of the POMS-BI (Lorr & McNair, 1984)
2. Brown & Harris (1989)	1. Degree of devastation, three indices: number of rooms entered, property damaged, and property disarranged (ransacking)	1. Emotional reactions to the burglary, composite score
	2. Value of stolen goods, three indices: primarily sentimental, primarily monetary, or both sentimental and monetary	2. Feelings of safety in the home, neighborhood, and the city (composite score)
	3. Satisfaction with police response, three indices: satisfaction with response time (i.e., time elapse since victim call), satisfaction with procedures (i.e., recording facts and taking evidence), and satisfaction with sensitivity (i.e., treatment of victims’ feelings)	
	4. Social coping, two indices: a composite score of talking to someone within 24 hours after the burglary and a composite score of the perceived effectiveness of this strategy (in terms of feeling better)	
	5. Neighbor-oriented coping, two indices: a composite score of neighbor-oriented behavior changes and a composite score of the perceived effectiveness of this strategy (in terms of feeling more secure)	
	6. Self-oriented coping, two indices: a composite score of self-oriented behavior changes and a composite of the perceived effectiveness of this strategy (in terms of feeling more secure)	
3. Chung et al. (2014)	1. The burglary experience, 11 aspects: number of months since burglary, number of months living in burgled house, still living in burgled house, planning to move, burglary committed through forced entry, the total value of lost belongings, loss of belongings with sentimental value, being at home during burglary, no previous burglary experience, moved as a result of burglary, damage of property	1. PTSD symptom level as measured by the IES (Horowitz, Wilner, & Alvarez, 1979)
	2. Personality traits as measured by the 48-item version of the EPQ (Eysenck & Eysenck, 1991), 3 types: extraversion, psychoticism, neuroticism	2. Mental health problems as measured by the 28-item version of the GHQ (Goldberg & Hillier, 1979)
	3. Coping strategies as measured by the WOC (Folkman & Lazarus, 1998), two types: emotion-focused coping and problem-focused coping	
4. Kobayashi (1996)	1. Age	1. Fear of revictimization, composite score
	2. Male sex	2. An increase in psychological symptoms, composite score
	3. Precautions taken prior to burglary, composite score	
	4. Closeness of relations with family and neighbors, composite score	
	5. Perceived police help, composite score	
	6. Wrong image of burglars, composite score	
	7. Fear of revictimization, composite score	
5. Kunst et al. (2013)	1. Age	1. PTSD symptom number at time 1 as measured by the TSQ (Brewin et al., 2002)
	2. Female sex	2. PTSD symptom level at time 2 as measured by the (Foa et al., 1993)
	3. At home during burglary	
	4. Recalled peritraumatic distress as measured by the PDI (Brunet et al., 2001)	
	5. Satisfaction with police performance (e.g., police politeness), composite score	
	6. Satisfaction with police procedure (e.g., police efficiency), composite score	
	7. PTSD symptoms at time 1 as measured by the TSQ (Brewin et al., 2002)	
6. Maguire (1980)	1. Female sex	1. Severity of psychological impact of burglary on victim according to 10 persons instructed to read each victim’s account of the burglary’s effects (more serious vs. less serious effects)
	2. Female victims’ marital status (married, single, separated/divorced, widowed)	
	3. Female victims’ social status (working class versus middle class)	
	4. Female victims’ living status (living alone versus living with others)	
	5. Burglary in female’s house committed during night-time	
	6. Female being at home during burglary	
	7. Burglary in female’s house committed through forced entry	
7. Mawby et al. (1999)	1. Country (United Kingdom, Poland, or Hungary)	1. Being affected by the burglary as measured by a single item with 5 response categories (“very much affected,” “quite a lot,” “a little,” “not at all,” “other”)
	2. Female sex	
	3. Prosperity as indicated by not having a car or not having been on holiday recently	
	4. Not being insured	
8. Waller & Okihiro (1978)	1. Male sex	1. Emotions experienced immediately after confrontation with the burglar or discovery of the burglary, six types: surprise, fear, anger, upset, relaxed, calm
	2. Planning to live long in burgled house	2. Fear of being alone
	3. Having made major alterations to burgled house	3. Fear of entering one’s house or rooms within one’s house
	4. Desire to see burglar imprisoned	
9. Winkel & Vrij (1993) and Winkel, Denkers, & Vrij (1994)	1. Internal attribution style, two indices: behavior attribution (i.e., attributing positive or negative events to one’s own behavior) and character attribution (i.e., attributing positive or negative events to one’s own character)	1. Fear of crime inside the house
	2. External attribution style (i.e., attributing positive or negative events to external causes)	2. Fear of crime outside the house
		3. Fear of crime inside and outside the house combined
10. Wylie (1993)	1. Age group (15–24 years, 25–39 years, 40–59 years, and 60 or older)	1. Immediate emotions (i.e., experienced in the first 24 hours after the burglary), composite score of 10 possible emotions: angry, fear, calm, anxious, shocked, depressed, numb, guilty, sad, and insecure
	2. Marital status (never married, married, divorced, separated, widowed)	
	3. Living alone	
	4. Social support received during the last month, composite score	
	5. Presence of other life stressors	
	6. Insurance status (full cover, partial cover, no insurance)	2. Long-term emotions (i.e., experienced at the time of the interview), composite score of 10 possible emotions: angry, fear, calm, anxious, shocked, depressed, numb, guilty, sad, and insecure
	7. Previous burglary	
	8. Monetary value of loss (in terms of approximate replacement value)	
	9. Sentimental value of loss	
	10. Degree of disarrangement	
	11. Degree of territorial intrusion as calculated by the product of the number of areas from which goods were stolen and/or which were disarranged during the burglary and the relative importance of these rooms for the victim	3. Intrusion symptom level, composite score
	12. Police handling of burglary (through telephone contact only vs. through a house visit)	

EPQ = Eysenck Personality Questionnaire; GHQ = General Health Questionnaire; IES = Impact of Events Scale; PDI = Peritraumatic Distress Inventory; POMS-BI = Profile of Moods States-Bipolar Form; PSS-SR = Self-Report Version of the PTSD Symptom Scale; PTSD = posttraumatic stress disorder; TSQ = Trauma Screening Questionnaire; WOC = Ways of Coping Checklist.

Table 2 provides an overview of the studies’ methodological qualities according to the Cambridge Quality Checklists. As shown in Table 2, all studies scored rather low on these checklists. The scores for the correlate subscale varied between 0 and 2. This means that all included studies scored poor in terms of sampling method, response rate, sample size, and/or reliability of the indices used to measure the correlate(s) of interest. The scores on the risk factor subscale varied between 1 and 3, with most studies scoring either 1 or 2 on the indices used to measure the outcome(s) of interest, meaning that they were either cross-sectional or retrospective in nature. The scores on the checklist for causal risk factors varied between 2 and 5, with most studies scoring 2 for at least one outcome of interest. This was due to the fact that none of the studies tested whether variation in the risk factor(s) of interest was associated with change in the study outcome(s).

**Table 2.** Study Quality According to Cambridge Quality Checklists*.

	Checklist for Correlates					Checklist for Risk Factors†	Checklist for Causal Risk Factors†
Authors	Sampling Method	Response Rate	Sample Size	Measurement of Correlate(−s)†	Measurement of Outcome(−s)†		
1. Beaton et al. (2000)	0	0	0	1:0	1:0 2:0	1:1	1:2 2:2
2. Brown & Harris (1989)	0	0	0	1:0 2:0 3:0 4:0 5:0 6:0	1:0 2:0	1:2 2:2 3:1 4:2 5:2 6:2	1:2 2:2
3. Chung et al. (2014)	0	0	0	1:0 2:1 3:1	1:1 2:1	1:2 2:1 3:1	1:5 1:5
4. Kobayashi (1996)	0	0	0	1:0 2:0 3:0 4:0 5:1 6:0 7:0	1:0 2:1	1:1 2:1 3:2 4:2 5:2 6:1 7:1	1:5 2:5
5. Kunst et al. (2013)	0	0	0	1:0 2:0 3:0 4:1 5:1 6:1 7:1	1:1 2:1	1:1/3‡ 2:1/3‡ 3:2/3‡ 4:2/3‡ 5:1/3‡ 6:1/3‡ 7:3	1:2 2:5
6. Maguire (1980)	0	0	0	1:0 2:0 3:0 4:0 5:0 6:0 7:0	1:0	1:1 2:1 3:1 4:1 5:2 6:2 7:2	1:2
7. Mawby et al. (1999)	0	0	0	1:0 2:0 3:0 4:0	1:0	1:1 2:1 3:1 4:1	1:2
8. Waller & Okihiro (1978)	1	0	0	1:0 2:0 3:0 4:0	1:0 2:0 3:0	1:1 2:1 3:2 4:1	1:2 2:2 3:2
9. Winkel & Vrij (1993); Winkel et al. (1994)	0	0	0	1:0 2:0	1:1 2:1 3:0	1:1 2:1	1:2 2:2 3:5
10. Wylie (1993)	0	1	0	1:0 2:0 3:0 4:0 5:0 6:0 7:0 8:0 9:0 10:0 11:0 12:0	1:0 2:0 3:0	1:1 2:1 3:1 4:2 5:1 6:1 7:2 8:2 9:2 10:2 11:2 12:2	1:2 2:2 3:2

* There are three checklists:

1. Checklist for correlates (five items):

- Sampling method: 1 = total population sampling or random sampling, 0 = convenience sampling or case control sampling.

- Response rate: 1 = response and retention rates ≥ 70% and differential attrition ≤ 10%, 0 = response rate < 70%, or retention rate < 70, or differential attrition > 10%.

- Sample size: 1 = sample size ≥ 400, 0 = sample size < 400.

- Measurement of the correlate: 1 = reliability coefficient ≥ .75 and reasonable face validity, or criterion or convergent validity coefficient ≥ .3, or more than one instrument or information source used to assess correlate, 0 = none of the aforementioned.

- Measurement of the outcome: 1 = reliability coefficient ≥ .75 and reasonable face validity, or criterion or convergent validity coefficient ≥ .3, or more than one instrument or information source used to assess outcome, 0 = none of the aforementioned.

2. Checklist for risk/protective factors (1 item): 1 = cross-sectional data, 2 = retrospective data, 3 = prospective data.

3. Checklist for causal risk/protective factors (1 item): 1 = study with no comparison group—no analysis of change, 2 = inadequately controlled study—no analysis of change, 3 = uncontrolled study—with analysis of change, 4 = inadequately controlled study—with analysis of change, 5 = controlled non-experimental study—no analysis of change, 6 = controlled non-experimental study—with analysis of change, and 7 = randomized experiment targeting a risk factor.

† Numbers before colons refer to the correlates and outcomes described in Table 1.

‡ For these correlates, scores differed between the first and the second outcome.

### Factors Investigated as Correlates of Psychological Distress

#### Pre-burglary factors

This category of factors was further divided into three subcategories: sociodemographic factors, personality-related factors, and a rest category of “other” pre-burglary factors.

##### Sociodemographic factors

This subcategory included the factors sex, age, country of origin, marital status, closeness of relations with family and neighbors, living alone, belonging to the working class/middle class, prosperity, and insurance status.

*Sex.* This factor was investigated as a potential determinant of psychological distress in six studies. Maguire (1980) found that female victims experienced more serious effects from the burglary than male victims, and Mawby et al. (1999) found that female victims were more often “very much affected” by the burglary than male victims. Mixed results were obtained by the four remaining studies. Beaton et al. (2000) found that female victims experienced more mental health problems than male victims. However, they only found this for mental health problems assessed between 7 and 12 days after the burglary and not for mental health problems assessed 4–5 weeks later. They also found that female victims were more anxious, tired, unsure, and confused than male victims when interviewed between 7 and 12 days after the burglary, but not more hostile or depressed, and that they were more anxious, tired, and unsure than male victims when interviewed 4–5 weeks later, but not more hostile, depressed, or confused. Kobayashi (1996) found that male sex was negatively associated with fear of revictimization, though not with an increase in psychological symptoms. Kunst et al. (2013) found that female victims experienced more PTSD symptoms within 1 month after the burglary, but not within 1 month after the first assessment. Finally, Waller and Okihiro (1978) found that male victims experienced less fear after confrontation with the burglar or immediately after discovery of the burglary, less fear of being alone, and less fear of entering their house or entering rooms within their house than female victims, but they also found that male victims did not experience more or less surprise or anger after confrontation with the burglar or immediately after discovery of the burglary than female victims.

*Age.* This factor was investigated as a potential determinant of psychological distress in two studies. Wylie (1993) found that victims between 40 and 59 years experienced more emotions during the first 24 hours after the burglary than victims between 25 and 39 years, but this researcher did not detect any other age-group differences for this type of emotions and neither for emotions experienced in the long run or for level of intrusion symptoms (Wylie, 1993). Similarly, Kunst et al. (2013) found no association between age and PTSD symptoms, neither for PTSD symptoms measured within 1 month after the burglary nor for PTSD symptoms measured within 1 month after the first assessment.

*Country of origin.* This factor was investigated as a potential determinant of psychological distress by Mawby et al. (1999). The researchers found that victims from Poland and Hungary experienced higher levels of distress than victims from the United Kingdom (Mawby et al., 1999).

*Marital status.* This factor was investigated as a potential determinant of psychological distress in two studies. Maguire (1980) found that victims who were separated, divorced, or widowed experienced more serious effects from the burglary than victims who were single or married. However, the significance of this difference was only tested for female participants. A seemingly contrasting observation was made by Wylie (1993), who found that marital status was associated neither with emotions experienced during the first 24 hours after the burglary nor with emotions experienced in the long run or with level of intrusion symptoms.

*Closeness of relations with family and neighbors.* This factor was investigated as a potential determinant of psychological distress by Kobayashi (1996). The researcher found that closeness of relations with family and neighbors was not associated with victims’ fear of revictimization or with an increase in psychological symptoms.

*Living alone.* This factor was investigated as a potential determinant of psychological distress by Wylie (1993). The researcher did not find any differences in emotions experienced during the first 24 hours after the burglary between victims who lived alone and those who did not and nor in emotions experienced in the long run or in level of intrusion symptoms.

*Belonging to the working class/middle class.* This factor was investigated as a potential determinant of psychological distress by Maguire (1980). The researcher found that victims belonging to the working class did not experience more or less serious effects from the burglary than victims belonging to the middle class. However, the significance of this difference was only tested for female participants.

*Prosperity.* This factor was investigated as a potential determinant of psychological distress by Mawby et al. (1999). The researchers used two indices to measure this factor: not having a car or not having been on holiday recently. They found that victims who did not own a car or who had not been on holiday recently were more often “very much affected” by the burglary than victims who did own a car or who had been on holiday recently.

*Insurance status.* This factor was investigated as a potential determinant of psychological distress in two studies. Mawby et al (1999) found that victims who were not insured were more often “very much affected” by the burglary than victims who were insured. A seemingly contrasting observation was made by Wylie (1993), who found that insurance status was associated neither with emotions experienced during the first 24 hours after the burglary nor with emotions experienced in the long run or with level of intrusion symptoms.

##### Personality-related factors

This subcategory included the factors personality traits, coping styles, and attribution styles.

*Personality traits.* This factor was investigated as a potential determinant of psychological distress by Chung et al. (2014). The researchers made a distinction between three personality traits: psychoticism, extraversion, and neuroticism. They found that extraversion and psychoticism but not neuroticism were associated with higher levels of PTSD symptoms and that psychoticism and neuroticism but not extraversion were associated with more mental health problems.

*Coping styles.* This factor was investigated as a potential determinant of psychological distress in two studies. Brown and Harris (1989) assessed three types of coping: social coping, neighborhood-oriented coping, and self-oriented coping. Each type of coping was measured with two indices. Social coping was operationalized as “talking to someone within 24 hours after the burglary” and as “the perceived effectiveness of talking to someone as a coping strategy”, neighborhood-oriented coping was operationalized as “neighbor-oriented behavior changes” and as “the perceived effectiveness of neighbor-oriented behavior changes as coping strategies”, and self-oriented coping was operationalized as “self-oriented behavior changes” and as “the perceived effectiveness of self-oriented behavior changes as coping strategies”. The researchers found that self-oriented behavior changes were associated with more emotional reactions to the burglary. However, neither the perceived effectiveness of these behavior changes nor any of the social coping or neighbor-oriented coping strategies were associated with such reactions. Chung et al. (2014) assessed two types of coping: emotion-focused coping and problem-focused coping. They found that emotion-focused but not problem-focused coping was associated with higher levels of PTSD symptoms and that both emotion-focused and problem-focused coping was associated with more mental health problems.

*Attribution styles.* This factor was investigated as a potential determinant of psychological distress by Winkel et al. (1994) and Winkel and Vrij (1993). The researchers made a distinction between internal (behavior versus character) and external attribution styles. They found that victims with an external attribution style (i.e., those who attribute positive or negative events to external causes) reported more fear of crime outside the house and more fear inside the house than victims with a behavior attribution style (i.e., those who attribute positive or negative events to their own behavior), but they did not find any differences on these indices of psychological distress between victims with a character attribution style (i.e., those attribute positive or negative events to their own character) and victims with a behavior attribution style or victims with an external attribution style. When they analyzed their data through path analysis, the researchers found that having an external attribution style was associated with participants’ scores on a combined measure of fear inside and fear outside the house; a higher score on this coping style was associated with more fear (Winkel & Vrij, 1993; Winkel et al., 1994).

#### Other pre-burglary factors

This subcategory included the factors number of months living in burgled house, precautions taken prior to the burglary, wrong image of burglars, value of the home to the victim, life stressors, and previous domestic burglary victimization.

*Number of months living in burgled house.* This factor was investigated as a potential determinant of psychological distress by Chung et al. (2014). The researchers found that it was associated with more mental health problems, but not when adjusting for potential confounding by other factors. They did not find any associations between the number of months living in burgled house and PTSD symptom levels.

*Precautions taken prior to the burglary and wrong image of burglars.* These factors were investigated as potential determinants of psychological distress by Kobayashi (1996). The researcher did not find significant associations between these factors and fear of revictimization or an increase in psychological symptoms.

*Value of the home to the victim.* This factor was investigated as a potential determinant of psychological distress by Waller and Okihiro (1978). These researchers used two indices to measure this factor: planning to live long in the burgled house and having made major alterations to the burgled house. Psychological distress was measured in terms of emotions experienced immediately after confrontation with the burglar or discovery of the burglary, fear of being alone, and fear of entering one’s house or rooms within one’s house. Neither planning to live long in burgled house nor having made major alterations to the burgled house was associated with any of these outcomes.

*Life stressors.* This factor was investigated as potential determinants of psychological distress by Wylie (1993). The researcher found that it was associated neither with emotions experienced during the first 24 hours after the burglary nor with emotions experienced in the long run or with level of intrusion symptoms.

*Previous domestic burglary victimization.* This factor was investigated as a potential determinant of psychological distress in two studies. Wylie (1993) found that it was associated neither with emotions experienced during the first 24 hours after the burglary nor with emotions experienced in the long run or with level of intrusion symptoms, and Chung et al. (2014) found that it was associated neither with PTSD symptoms nor with mental health problems.

#### Peri-burglary factors

This category of factors was further divided into three subcategories: act-related factors, damage-related factors, and a rest category of “other” peri-burglary factors.

##### Act-related factors

This subcategory included the factors time of burglary, victim at home during the burglary, forced entry, and degree of territorial intrusion.

*Time of burglary*. This factor was investigated as a potential determinant of psychological distress by Maguire (1980). The researcher found that victims did not experience more serious effects from the burglary when the burglary had been committed during night-time than when it had been committed during the day. However, the significance of this difference was only tested for female participants.

*Victim at home during the burglary.* This factor was investigated as a potential determinant of psychological distress in three studies. Both Chung et al. (2014) and Kunst et al. (2013) found that victims did not experience more PTSD symptoms when the burglary had been committed while the victim was at home than when he or she was not at home during the burglary. Chung et al. (2014) further found that victims who had been at home during the burglary did not experience more mental health problems than those who had not been at home during the burglary. Similarly, Maguire (1980) found that victims did not experience more serious effects from the burglary when the burglary had been committed while the victim was at home than when he or she was not at home during the burglary, but the significance of this difference was tested for female participants only.

*Forced entry.* This factor was investigated as a potential determinant of psychological distress in two studies. Chung et al. (2014) found that it was associated with more mental health problems, but not when they adjusted for potential confounding by other factors. They did not find any association between forced entry and PTSD symptom levels. Likewise, Maguire (1980) found that victims did not experience more serious effects from the burglary when the burglary had been committed through forced entry than when it had not been committed through forced entry, though the significance of this difference was only tested among female participants.

*Degree of territorial intrusion.* This factor was investigated as a potential determinant of psychological distress by Wylie (1993) and refers to the number and importance of rooms stolen from or disarranged. The researcher found that it was associated with more emotions experienced during the first 24 hours after the burglary and higher levels of intrusion symptoms, but not with more or less emotions experienced in the long run.

##### Damage-related factors

This subcategory included the factors degree of devastation, degree of disarrangement, and value of stolen goods.

*Degree of devastation.* This factor was investigated as a potential determinant of psychological distress in two studies. Brown and Harris (1989) used three indices to measure this factor: number of rooms entered, property damaged, and property disarranged (i.e., ransacked). All three were associated with more emotional reactions to the burglary. However, while the first two were also associated with feelings of safety, the third was not. Slightly different results were obtained by Chung et al. (2014). These researchers only tested the significance of the associations between damage of property and the outcomes of interest. They found that damage of property was associated with more mental health problems, but not when adjusting for potential confounding by other factors, and neither did they find an association between damage of property and PTSD symptom levels.

*Degree of disarrangement.* This factor was investigated as a potential determinant of psychological distress by Wylie (1993). The researcher found that this factor was associated with more emotions experienced during the first 24 hours after the burglary and higher levels of intrusion symptoms, but not with more or less emotions experienced in the long run.

*Value of stolen goods.* This factor was investigated as a potential determinant of psychological distress in three studies. All studies used two indices to measure this factor: sentimental and monetary value of loss. The three studies yielded mixed results. Brown and Harris (1989) found that a combination of sentimental and monetary value of stolen goods was associated with more feelings of safety, but not with more or less emotional reactions to the burglary. A primarily sentimental value of stolen goods or a primarily monetary value of stolen goods was associated neither with emotions to the burglary nor with feelings of safety. Chung et al. (2014) found that the loss of belongings with sentimental value but not the total value of lost belongings was associated with higher levels of PTSD symptoms. This association remained significant when the researchers adjusted for potential confounding by other factors. On the other hand, neither the total value of lost belongings nor loss of belongings with sentimental value was associated with mental health problems. Finally, Wylie (1993) found that sentimental value of loss was associated with more experienced emotions, both during the first 24 hours after the burglary and in the long run, and with higher levels of intrusion symptoms. Monetary value of loss was associated with more experienced emotions during the first 24 hours after the burglary and with higher levels of intrusion symptoms, but not with more or less emotions experienced in the long run.

##### Other peri-burglary factors

This subcategory included only the factor recalled peritraumatic distress.

*Recalled peritraumatic distress.* This factor was investigated as a potential determinant of psychological distress by Kunst et al. (2013). The researchers found that it was associated with more PTSD symptoms, both within 1 month after the burglary and within 1 month after first assessment.

#### Post-burglary factors

This category was further divided into two subcategories: police response-related factors and a rest category of “other” post-burglary factors.

##### Police response-related factors

This subcategory included the factors satisfaction with the police response, perceived police help, and police handling of burglary.

*Satisfaction with the police response.* This factor was investigated as a potential determinant of psychological distress in two studies. Both studies obtained mixed results. Brown and Harris (1989) used three indices to measure this factor: satisfaction with response time (i.e., time elapse since victim call), satisfaction with procedures (i.e., recording facts and taking evidence), and satisfaction with sensitivity (i.e., treatment of victims’ feelings). All three were associated with more emotional reactions to the burglary. However, while the first two were also associated with feelings of safety, the third was not. Kunst et al. (2013) used two indices to assess satisfaction with the police response: satisfaction with police procedure (e.g., police politeness) and satisfaction with police performance (e.g., police efficiency). The researchers found that both factors were not associated with PTSD symptoms, neither within 1 month after the burglary nor within 1 month after first assessment. There were, however, two exceptions to these findings: victims who experienced a high number of PTSD symptoms within 1 month after the burglary experienced higher levels of PTSD symptoms within 1 month after first assessment if they had reported lower levels of satisfaction at first assessment.

*Perceived police help.* This factor was investigated as a potential determinant of psychological distress by Kobayashi (1996). The researcher found that it was not associated with fear of revictimization or an increase in psychological symptoms.

*Police handling of burglary.* This factor was investigated as a potential determinant of psychological distress by Wylie (1993). The researcher found that victims did not experience more emotions when the police had handled the burglary through a telephone contact than when they had handled it through a house visit, neither during the first 24 hours after the burglary nor in the long run. On the other hand, they did experience higher levels of intrusion symptoms when the police had handled the burglary through telephone contact than when they had handled it through a house visit.

##### Other post-burglary factors

This subcategory included the factors number of months since burglary, still living in burgled house, planning to move, moved as a result of the burglary, desire to see burglar imprisoned, social support, and fear of revictimization.

*Number of months since burglary, still living in burgled house, planning to move, and moved as a result of the burglary.* These factors were investigated as potential determinants of psychological distress by Chung et al. (2014). The researchers found that none of these factors were associated with PTSD symptoms or mental health problems.

*Desire to see burglar imprisoned.* This factor was investigated as a potential determinant of psychological distress by Waller and Okihiro (1978). The researchers found that this factor was not associated with emotions experienced immediately after confrontation with the burglar or discovery of the burglary and neither with fear of being alone or fear of entering one’s house or rooms within one’s house.

*Social support.* This factor was investigated as a potential determinant of psychological distress by Wylie (1993). The researcher did not find a significant association between this factor and experienced emotions, neither during the first 24 hours after the burglary nor within the long run, or with intrusion symptoms.

*Fear of revictimization.* This factor was investigated as a potential determinant of psychological distress by Kobayashi (1996). The researcher found that this factor was associated with an increase in psychological symptoms.

### Synthesis

Due to the heterogeneity in study designs and the poor methodological qualities and inconsistency in findings, it is too early to draw definite conclusions about determinants of psychological distress in domestic burglary victims on the basis of the reviewed studies. Nevertheless, taken together, we think their results preliminary indicate that three factors should be considered as potential risk or protective factors of psychological distress. First, it seems important to consider sex as a potential risk/protective factor for psychological distress. Six studies investigated this factor as a potential determinant of psychological distress (Beaton et al., 2000; Kobayashi, 1996; Kunst et al., 2013; Maguire, 1980; Mawby et al., 1999; Waller & Okihiro, 1978). All these studies found that female sex was associated with higher levels of psychological distress, though not for all the indices they used to measure this outcome. Second, it seems important to consider damages caused by the burglary as a potential risk factor for psychological distress. This factor was investigated by three studies and all of them found a significant association for at least one of the indices they used to measure psychological distress (Brown & Harris, 1989; Chung et al., 2014; Wylie, 1993). Third, it seems important to consider victims’ satisfaction with the police response as a factor that might protect victims against psychological distress. The association between this factor and psychological distress was investigated by only two studies (Brown & Harris, 1989; Kunst et al., 2013), but both found it to be significant and one of them was the only study which used a prospective design (Kunst et al., 2013).

Apart from preliminarily indicating that female sex, damages caused by the burglary, and satisfaction with the police response are determinants of psychological distress, the review findings also suggest that it is important to make a distinction between screening for psychological distress experienced in the immediate aftermath of the burglary and screening for psychological distress experienced in the long run. Two studies assessed the same indices of psychological distress at two time points and found that risk or protective factors’ associations with psychological distress differed between the two time points (Beaton et al., 2000; Kunst et al., 2013). For example, Beaton et al. (2000) found that female sex was associated with feeling confused between 7 and 12 days after the burglary, but not between 4 and 5 weeks later. Two other studies did not assess psychological distress at different time points but distinguished between currently experienced distress or long-term distress and recollections of distress experienced immediately or shortly after the burglary and found that these outcomes were differently associated with the risk and protective factors they investigated (Waller & Okihiro, 1978; Wylie, 1993). For example, Wylie (1993) found that the degree of territorial intrusion was associated with more experienced emotions during the first 24 hours after the burglary, but not with emotions experienced in the long run and neither with current symptoms of intrusion.

## Discussion

This study aimed to identify potential determinants of psychological distress in domestic burglary victims. This was deemed important, as many countries are obliged to screen crime victims who report the crime to the police or another law enforcement agency. As mentioned in the synthesis section, the reviewed studies were mostly outdated and do not allow for drawing definite conclusions about factors that put domestic burglary victims at risk for or protect them against psychological distress. Nevertheless, they preliminarily indicate that it is important to use female sex, damages caused by the burglary, and dissatisfaction with the police response as red flags in victim screening procedures.

The finding that female sex is associated with higher levels of psychological distress after domestic burglary victimization is in line with a large body of research about sex differences in trauma outcome exposure (see Breslau, 2009; Olff et al., 2007; Tolin & Foa, 2006). Currently, little is known about possible explanations for this between-sexes difference in psychological distress. On the basis of a systematic literature review, Christiansen and Berke (2020) have recently argued that these differences are due to genetic predispositions and hormones which are more prevalent among women than in men, but whether this also applies to victims of domestic burglary victimization is unknown. An alternative explanation might be that victimization experiences have another meaning for women than for men. With regard to domestic burglary, it has been hypothesized that women fear this type of crime more than men because they think a confrontation with the burglar puts them at risk of sexual assault (e.g., Hirtenlehner & Farrall, 2014; Mellgren & Ivert, 2019). As a consequence, the level of distress experienced during domestic burglary may be much higher for female than for male victims. If so, this puts them at a higher risk of developing psychological distress in the aftermath of the burglary than men, because distress experienced *during* potentially traumatic events is one of the strongest predictors of experiencing psychological distress in their aftermath (Thomas et al., 2012; Vance et al., 2018). Preliminary evidence for this possibility was provided by the Kunst et al. (2013) study, which found that females had experienced higher levels of peritraumatic distress during the burglary than men.

The apparent association between satisfaction with the police response suggests that the police may play an important role in victims’ psychological recovery from the burglary. This is in line with the *Theory of Therapeutic Jurisprudence*, which posits that people can either benefit or suffer from their involvement in legal procedures and contacts with legal actors, such as law enforcement professionals (Wexler, 1990). It also fits with previous research on the impact of crime victims’ evaluations of their involvement in criminal proceedings. Studies addressing this topic have shown that more positive evaluations are associated with higher levels of psychological well-being, though particularly during the first few weeks after the crime (see Kunst et al., 2015). This is understandable given the fact that most victims return to their pre-crime level of psychological well-being during this period (cf. Kunst & Koster, 2017; Walters et al., 2007; T. D. Wohlfarth et al., 2003; T. Wohlfarth et al., 2002). On the other hand, it seems important to acknowledge that the association between crime victims’ evaluations of the police response and the degree of psychological distress they experience may be confounded by other factors. A potential confounder might be the extent to which crime victims feel connected to others within their family and community; victims who feel connected to (significant) others may not need the police to feel acknowledgment and support (Barkworth & Murphy, 2016). Preliminary evidence for this contention was recently provided by Avery et al. (2020), who found that the size of the association between participants’ perceptions of police effectiveness and levels of psychological distress decreased when they adjusted for participants’ perceptions of social cohesion in their neighborhood.

The observed association between damages caused by the burglary and psychological distress is in line with Harris and Brown’s (1989) hypothesis that intrusions of primary territories are potentially traumatic, but it does not provide insight into factors that can explain why this is so. According to Brown and Harris (1989), such intrusions may shatter one’s belief in the inviolability of his/her primary territory, but none of the three studies which investigated damages caused by the burglary as determinants of psychological distress tested whether and to what extent participants’ beliefs in the inviolability of their primary territory were shattered by these damages and neither whether and to what extent this shattering (statistically) mediated or moderated the association between damages and psychological distress. This limitation, unfortunately, also applies to studies which used the theory of shattered assumptions as a framework to investigate the psychological impact of other types of crime victimization than domestic burglary victimization; most of these studies did not test all the premises of this theory (e.g., Borwell et al., 2021; Denkers, 1996; Denkers & Winkel, 1998) and/or did not exclusively focus on crime victimization (e.g., Ferrajão & Elklit, 2020; Poulin & Silver, 2019; Schuler & Boals, 2016). To date, Norris and Kaniasty (1991) appear to be the only one to have tested the theory in its entirety. They found that both violent and property crime victimization were associated with higher levels of psychological distress than non-victimization and that these associations were partly mediated by a shattering of victims’ beliefs about their own safety. These findings provide only preliminary evidence for the validity of the theory of shattered assumptions though, as they were based on a retrospective assessment of crime victimization and prior beliefs.

### Directions for Future Research

Given the preliminary status of this review’s findings, it is important that future studies do more and better research into factors that may predispose victims of domestic burglary to or protect them against psychological distress. Our findings suggest that victim sex, damages caused by the burglary, and victims’ evaluations of the police response are important factors to consider as potential determinants of psychological distress, but it is just as important to consider other possible predictors of psychological distress. After all, the existing studies investigated a rather narrow array of risk and protective factors. For example, none of them investigated whether psychological distress among domestic burglary victims varied by race or ethnicity or sexual orientation. The meaning of domestic burglary victimization may differ according to these person characteristics.

Furthermore, it is important that future studies more often use prospective designs and validated instruments to investigate associations between potential risk factors and psychological distress. After all, only when the risk or protective factor of interest is measured before the outcome of interest and when this outcome is properly operationalized, is it justified to speak of a “risk” or “protective” factor of that particular outcome (cf. Kraemer et al., 2001).

Finally, to explain why certain factors render victims at risk of or protect them against psychological distress, it is necessary that they base their investigation on Harris and Brown’s (1989) integration of Janoff-Bulman’s shattered assumptions theory and Altman’s (1975) concept of territoriality. For example, they should test whether any observed relation between victim sex and psychological distress is confounded by within-sex differences in beliefs about the inviolability of their homes. It will not be easy to test this properly, as a prospective study design is required to see whether victims’ beliefs have changed after the burglary. Preferably, this is done in a series of replications within and across countries to ensure that findings are not biased by between-location differences in relevant risk and protective factors of psychological distress. Such an approach costs a lot of money and time, but is indispensable if we want to identify victims in need of professional support services and fulfill national and international legal obligations in terms of screening and referral (Winkel et al., 2003). In view of the large numbers of burglaries committed each year, these costs should not hold back conducting prospective research among domestic burglary victims.

### Study Limitations and Strengths

Before concluding, we should mention two important limitations of our study: it is based on studies published in English and included in traditional scholarly literature databases. Our findings and conclusions may therefore suffer from an English language and a peer-reviewed journal bias. Nevertheless, these limitations do not take away from the importance of our study. After all, it was the first to systematically review the literature on determinants of psychological distress among domestic burglary victims. It was therefore able to provide an overview of the state of the art across countries and identify gaps that require further research.

## Conclusion

Domestic burglary victimization is a potentially traumatic experience. To identify those in need of professional help services, it is necessary that law enforcement and victim support agencies know which factors put victims at risk of or protect them against psychological distress. Unfortunately, this review suggests that currently very little is known about the determinants of psychological distress in domestic burglary victims and that additional research is necessary to fill this gap in the literature (see Table 3).

**Table 3.** Critical Findings.

Very few studies have investigated which factors render victims of domestic burglary at risk of or protect them against experiencing or developing psychological distress
The few studies that did investigate risk and protective factors of psychological distress in domestic burglary victims are rather old, have poor methodological quality, and pay little attention to theory
Available findings suggest that victim sex, damages caused by the burglary, and evaluations of the police response are determinants of psychological distress among domestic burglary victims

## Supplemental Material

sj-docx-1-tva-10.1177_15248380231155525 – Supplemental material for Psychological Distress Among Domestic Burglary Victims: A Systematic Review of Possible Risk and Protective Factors Click here for additional data file. Supplemental material, sj-docx-1-tva-10.1177_15248380231155525 for Psychological Distress Among Domestic Burglary Victims: A Systematic Review of Possible Risk and Protective Factors by Maarten Kunst and Dante Hoek in Trauma, Violence, & Abuse

## Notes

1. Interested readers may request the PRISMA protocol from the authors.

2. The exact search strings differed slightly depending on the search field and the functionality of the literature base. They can be obtained from the authors.

3. Levels 2, 3, 4, 6, and 7 are the equivalents of the Maryland Scientific Methods Scale levels 1, 2, 3, 4, and 5, respectively (Murray et al., 2009).
