# Supplementary material for: Psychological Distress Among Domestic Burglary Victims: A Systematic Review of Possible Risk and Protective Factors
Source: Trauma Violence Abuse. 2023 Feb 27;25(1):430–47. doi: 10.1177/15248380231155525 (PMC10666482; doi:10.1177/15248380231155525)
Supplement: sj-docx-1-tva-10.1177_15248380231155525 – Supplemental material for Psychological Distress Among Domestic Burglary Victims: A Systematic Review of Possible Risk and Protective Factors [file sj-docx-1-tva-10.1177_15248380231155525.docx]

Appendix 1

*Summary of included studies*

| Study | Location | Sample characteristics | Assessment characteristics | Risk/protective factors | Psychological distress | Results |
| --- | --- | --- | --- | --- | --- | --- |
| 1. Beaton, Cook, Kavanagh, & Herrington (2000) | UK, town in South Wales | 20 domestic burglary victims (12 males, 8 females) | Questionnaires administrated during two home visits conducted by a victim support volunteer (one between 7 and 12 days after the burglary and one 4 to 5 weeks later) | 1. Female sex | 1. Mental health problems as measured by the 12-item version of the General Health Questionnaire (Goldberg, 1978) 2. Mood states as measured by the six subscales (Composed-Anxious, Agreeable-Hostile, Elated-Depressed, Confident-Unsure, Energetic-Tired, and Clear-headed - Confused) of the Profile of Moods States - Bipolar Form (POMS-BI; Lorr & McNair, 1984) | 1. Mental health problems:  - bivariate analysis: female victims reported more mental health problems than male victims, but only at time 1 - multivariate analysis: not performed - path analysis: not performed  1. Mood states:  - bivariate analysis: female victims scored higher on the POMS-BI than males, but at time 1 only on the Composed-Anxious, Energetic-Tired, Confident-Unsure, and Clear-headed – Confused subscales and at time 2 only on the Composed-Anxious, Energetic-Tired, and Confident-Unsure subscales - multivariate analysis: not performed - path analysis: not performed |
| 1. Brown & Harris (1989) | USA, Utah, Fort Worth | 44 domestic female burglary victims | Telephone interview conducted by a trained interviewer (between 1 and 37 months after the burglary) | 1. Degree of devastation, three indices: number of rooms entered, property damaged, and property disarranged (ransacking) 2. Value of stolen goods, three indices: primarily sentimental, primarily monetary, or both sentimental and monetary 3. Satisfaction with police response, three indices: satisfaction with response time (i.e. time elapse since victim call), satisfaction with procedures (i.e. recording facts and taking evidence), and satisfaction with sensitivity (i.e. treatment of victims’ feelings) 4. Social coping, two indices: a composite score of talking to someone within 24 hours after the burglary and a composite score of the perceived effectiveness of this strategy (in terms of feeling better) 5. Neighbor-oriented coping, two indices: a composite score of neighbor-oriented behavior changes and a composite score of the perceived effectiveness of this strategy (in terms of feeling more secure) 6. Self-oriented coping, two indices: a composite score of self-oriented behavior changes and a composite of the perceived effectiveness of this strategy (in terms of feeling more secure) | 1. Emotional reactions to the burglary, composite score 2. Feelings of safety in the home; neighbourhood; and the city, composite score | 1. Emotional reactions to the burglary:  - bivariate analysis:   - each index of degree of devastation was positively associated with emotional reactions to the burglary   - none of the indices of value of stolen goods was associated with emotional reactions to the burglary   - all indices of satisfaction with police response were negatively associated with emotional reactions to the burglary   - of all coping indices, only self-oriented behavior changes were associated with emotional reactions to the burglary; victims with higher scores on this index reported more emotional reactions to the burglary than those with lower scores - multivariate analysis: not performed - path analysis: not performed  1. Feelings of safety:  - bivariate analysis:   - of the three indices of degree of devastation, only range of rooms entered and property disarranged were associated with feelings of safety; victims with higher scores on these indices reported less feelings of safety than those with lower scores   - of the three indices of value of stolen goods, only both sentimental and monetary was associated with feelings of safety; victims with higher scores on this index reported less feelings of safety than those with lower scores   - of the three indices of satisfaction with police response, only satisfaction with response time and satisfaction with procedures were associated with feelings of safety; victims with higher scores on these indices reported more feelings of safety than those with lower scoresof all coping indices, only self-oriented behavior changes were associated with emotional reactions to the burglary; victims with higher scores on this index reported less feelings of safety than those with lower scores - multivariate analysis: not performed - path analysis: not performed |
| 1. Chung, Stedman, Hall, Marks, Thorhnhill, & Mehrshahi (2014) | UK, not further specified | 125 victims of domestic burglary (63 males, 62 females) | Questionnaire administrated during home visit conducted by research assistants (on average 11.4 months since the burglary) | 1. The burglary experience, 11 aspects: number of months since burglary, number of months living in burgled house, still living in burgled house, planning to move, burglary committed through forced entry, the total value of lost belongings, loss of belongings with sentimental value, being at home during burglary, no previous burglary experience, moved as a result of burglary, damage of property 2. Personality traits as measured by the 48-item version of the Eysenck Personality Questionnaire (EPQ, Eysenck & Eysenck, 1991), 3 types: extraversion, psychoticism, and neuroticism 3. Coping strategies as measured by the Ways of Coping Checklist (WOC; Folkman & Lazarus, 1998), two types: emotion-focused coping and problem-focused coping | 1. PTSD symptom level as measured by the Impact of Events Scale (IES; Horowitz, Wilner, & Alvarez, 1979) 2. Mental health problems as measured by the 28-item version of the General Health Questionnaire (Goldberg & Hillier, 1979) | 1. PTSD symptom level:  - bivariate analysis: of all aspects of the burglary experience, only the loss of belongings with sentimental value was associated with PTSD symptom levels; victims who had experienced such a loss reported higher levels of PTSD symptoms than those who had not^[[1]](#footnote-1)^ - multivariate regression analysis: when adjusting for personality traits and coping strategies, only loss of belonging with sentimental value, extraversion, psychoticism, and emotion-focused coping were associated with PTSD symptom level;   - victims who had experienced a loss of belongings with sentimental value reported higher levels of PTSD symptoms than those who had not;   - more extravert victims reported lower levels of PTSD symptoms than those less extravert;   - more psychotic victims reported lower levels of PTSD symptoms than those less psychotic;   - victims using more emotion-focused coping reported higher levels of PTSD symptoms than those using less emotion-focused coping - path analysis: not performed  1. Mental health problems:  - bivariate analysis: of all aspects of the burglary experience, only number of months since burglary, burglary committed through forced entry, and damage of property were associated with mental health problems; victims with these experiences reported more mental health problems than those without^[[2]](#footnote-2)^ - multivariate regression analysis: when adjusting for personality traits and coping strategies, only psychoticism, neuroticism, emotion-focused coping, and problem-focused coping were associated with mental health problems;   - more psychotic victims reported less mental health problems than those less psychotic   - more neurotic victims reported more mental health problems than those less neurotic;   - victims using more emotion-focused coping reported more mental health problems than those using less emotion-focused coping;   - victims using more problem-focused coping reported less mental health problems than those using less problem-focused coping - path analysis: not performed |
| 1. Kobayashi (1996) | Japan, not further specified | 322 victims of domestic burglary victims (111 males and 211 females) | Questionnaire completed briefly after the burglary (mostly 2 to 7 weeks later) | 1. Age 2. Male sex 3. Precautions taken prior to burglary, composite score 4. Closeness of relations with family and neighbors, composite score 5. Perceived police help, composite score 6. Wrong image of burglar, composite score 7. Fear of revictimization, composite score | 1. Fear of revictimization, composite score 2. An increase in psychological symptoms, composite score | 1. Fear of revictimization:  - bivariate analysis: not performed - multivariate regression analysis: when adjusting for the other risk/protective factors, only age and male sex were associated with fear of revictimization; older victims reported less fear of revictimization than younger victims and male victims reported less fear of revictimization than female victims - path analysis: not performed  1. An increase in psychological symptoms:  - bivariate analysis: not performed - multivariate regression analysis: when adjusting for the other risk/protective factors, only age was associated with an increase in psychological symptoms; older victims reported more increase in psychological symptoms than younger victims - path analysis: when testing for potential causality, only fear of revictimization was positively associated with an increase in psychological symptoms; victims who reported more fear of revictimization als reported more increase in psychological symptoms than those who reported less fear |
| 1. Kunst, Rutten, & Knijf (2013) | The Netherlands, several towns and villages Holland Midden police district | 95 victims of domestic burglary (32 males, 63 females) | 2 telephone interviews conducted by a research assistant (one within a month after the burglary and one within a month after the first interview) | 1. Age 2. Female sex 3. At home during burglary 4. Recalled peritraumatic distress as measured by the Peritraumatic Distress Inventory (PDI; Brunet et al., 2001) 5. Satisfaction with police performance (e.g. police politeness), composite score 6. Satisfaction with police procedure (e.g. police efficiency), composite score 7. PTSD symptoms at time 1 as measured by the Trauma Screening Questionnaire (TSQ; Brewin, Rose, Andrews, Green, Tata, McEvedy, & Foa, 2002) | 1. PTSD symptom number at time 1 as measured by the Trauma Screening Questionnaire (TSQ; Brewin, Rose, Andrews, Green, Tata, McEvedy, & Foa, 2002) 2. PTSD symptom level at time 2 as measured by the self-report version of the PTSD Symptom Scale (PSS-SR; Foa, Riggs, Dancu, & Rothbaum, 1993) | 1. PTSD symptom number at time 1:  - bivariate analysis: of all risk/protective factors, only female sex and recalled peritraumatic distress were associated with PTSD symptom numbers at time 1; - female victims reported more PTSD symptoms than male victims; - victims with higher levels of recalled peritraumatic distress reported more PTSD symptoms than those with lower levels - multivariate analysis: not performed - path analysis: not performed  1. PTSD symptom level at time 2:  - bivariate analysis: of all risk/protective factors, only recalled peritrauma distress and PTSD symptom number at time 1 were associated with PTSD symptom level at time 2; victims with higher levels of recalled peritraumatic distress and victims with more PTSD symptoms at time 1 reported higher PTSD symptom levels at time 2 - multivariate regression analysis: when adjusting for the other risk/protective factors, only recalled peritrauma distress, PTSD symptom number at time 1, and the interactions between PTSD symptom number at time 1 and satisfaction with police performance and between PTSD symptom number at time 1 and satisfaction with police procedure were associated with PTSD symptom level at time 2;   - victims with higher levels of recalled peritraumatic distress and more PTSD symptoms at time 1 reported higher PTSD symptom levels at time 2 than those with lower levels of recalled peritraumatic distress;   - victims with more PTSD symptoms at time 1 and a high level of satisfaction with police performance or procedure reported lower PTSD symptom levels at time 2 than those with more PTSD symptoms at time 1 and a low level of satisfaction with police performance or procedure - path analysis: not performed |
| 1. Maguire (1980) | United Kingdom, several towns and volleages in Thames Valley police district | 322 victims of domestic burglary (163 males and 159 females) | Face-to-face interview conducted in victims’ homes by the author and another researcher (between 4 and 10 weeks after the burglary) | 1. Female sex 2. Female victims’ marital status (married, single, separated/divorced, widowed) 3. Female victims’ social status (working class versus middle class) 4. Female victims’ living status (living alone versus living with others) 5. Burglary in female’s house committed during night-time 6. Female being at home during burglary 7. Burglary in female’s house committed through forced entry | 1. Severity of psychological impact of burglary on victim according to 10 persons instructed to read each victim’s account of the burglary’s effects (more serious versus less serious effects) | 1. Severity of psychological impact of burglary on victim  - bivariate analysis:   - in the entire sample female victims experienced more serious effects than male victims   - among female victims, marital status was the only factor associated with severity of psychological impact; victims who were separated, divorced, or widowed experienced more serious effects than those not belonging to any of these categories - multivariate analysis: not performed - path analysis: not performed |
| 1. Mawby, Gorgenyi, Ostrihanska, Walklate, & Wojcik (1999) | UK, Plymouth and Salford; Poland, Lublin and Warsaw; and Hungary, Miskolc | 200 victims of domestic burglary from Plymouth, Warsaw, and Lublin, 132 from Salford, and 77 from Miskolc (numbers of males and females not indicated) | Face-to-face interview conducted in victims’ homes by trained interviewers (either 6 to 8 weeks or 16 to 18 weeks after the burglary) | 1. Country (UK, Poland, or Hungary) 2. Female sex 3. Prosperity as indicated by not having a car or not having been on holiday recently 4. Not being insured | 1. Being affected by the burglary as measured by a single item with 5 response categories (“very much affected”, “quite a lot”, “a little”, “not at all”, “other”) | 1. Being affected by the burglary  - bivariate analysis: all risk/protective factores were associated with being affected by the burglary;   - victims from Poland and Hungary more often reported they were “very much affected” by the burglary than those from the UK;   - female victims more often reported they were “very much affected” by the burglary than male victims;   - victims who did not own a car more often reported they were “very much affected” by the burglary than those who did;   - victims who had not been on holiday recently more often reported they were “very much affected” by the burglary than those who had;   - victims who were not insured more often reported they were “very much affected” by the burglary than those who were - multivariate analysis: not performed - path analysis: not performed |
| 1. Waller & Okihiro (1978) | Canada, Toronto | 116 victims of domestic burglary (52 males, 62 females, 2 sex unknown) | Face-to-face interview conducted in victims' homes by trained interviewers (within 16 months after the burglary) | 1. Male sex 2. Planning to live long in burgled house 3. Having made major alterations to burgled house 4. Desire to see burglar imprisoned | 1. Emotions experienced immediately after confrontation with the burglar or discovery of the burglary, six types: surprise, fear, anger, upset, relaxed, calm 2. Fear of being alone 3. Fear of entering one’s house or rooms within one’s house | 1. Emotions experienced immediately after confrontation with the burglar or discovery of the burglary:  - bivariate analysis:   - male victims reported less fear after confrontation with the burglar or immediately after discovery of the burglary but not more or less surprise or anger than female victims^[[3]](#footnote-3)^   - planning to live long in burgled house and having made major alterations to burgled house were not associated with fear after confrontation with the burglar or immediately after discovery of the burglary^[[4]](#footnote-4)^   - desire to see burglar imprisoned was not associated with fear, anger, upset or surprise^[[5]](#footnote-5)^ - multivariate analysis: not performed - path analysis: not performed  1. Fear of being alone:  - bivariate analysis: male victims reported less fear of being alone than female victims^[[6]](#footnote-6)^ - multivariate analysis: not performed - path analysis: not performed  1. Fear of entering one’s house or rooms within one’s house:  - bivariate analysis: male victims reported less fear of entering their house or entering rooms within their house than female victims^[[7]](#footnote-7)^ - multivariate analysis: not performed - path analysis: not performed |
| 1. Winkel & Vrij (1993) and Winkel, Denkers, & Vrij (1994) | The Netherlands, province of Noord-Holland | 165 domestic burglary victims (101 males, 64 females) | Questionnaire completed briefly after the burglary (3 days later later) | 1. Internal attribution style, two indices: behavior attribution (i.e. attributing positive or negative events to one’s own behavior) and character attribution (i.e. attributing positive or negative events to one’s own character) 2. External attribution style (i.e. attributing positive or negative events to external causes) | 1. Fear of crime inside the house 2. Fear of crime outside the house 3. Fear of crime inside and outside the house combined | 1. Fear of crime inside the house:  - bivariate analysis:   - victims with an external attribution style reported more fear of crime inside the house than those with a behavior attribution style (bivariate analysis)   - victims with a character attribution style did not report more fear of crime inside the house than those with one of the two other attribution styles - multivariate analysis: not performed - path analysis: not performed  1. Fear of crime outside the house:  - bivariate analysis:   - victims with an external attribution style reported more fear of crime outside the house than those with a behavior attribution style   - victims with a character attribution style did not report more fear of crime outside the house than those with one of the two other attribution styles - multivariate analysis: not performed - path analysis: not performed  1. Fear of crime inside and outside the house combined:  - bivariate analysis: not performed - multivariate analysis: not performed - path analysis: when testing for potential causality, only external attribution style was associated with fear of crime inside and outside the house; victims using this attribution style to a larger extent scored higher on the combined score of fear inside and outside the house than those using this attribution style to a smaller extent |
| 1. Wylie (1993) | New Zealand, Canterbury Region, Christchurch | 102 female victims of domestic burglary | Face-to-face interviews conducted in the victim’s home or at the victim’s workplace by the author (between 8 and 11 weeks after the burglary) | 1. Age group (15 to 24 years, 25 to 39 years, 40 to 59 years, and 60 or older) 2. Marital status (never married, married, divorced, separated, widowed) 3. Living alone 4. Social support received during the last month, composite score 5. Presence of other life stressors 6. Insurance status (full cover, partial cover, no insurance) 7. Previous burglary 8. Monetary value of loss (in terms of approximate replacement value) 9. Sentimental value of loss 10. Degree of disarrangement 11. Degree of territorial intrusion as calculated by the product of the number of areas from which goods were stolen and/or which were disarranged during the burglary and the relative importance of these rooms for the victim 12. Police handling of burglary (through telephone contact only versus through a house visit) | 1. Immediate emotions (i.e. experienced in the first 24 hours after the burglary), composite score of 10 possible emotions: angry, fear, calm, anxious, shocked, depressed, numb, guilty, sad, and insecure 2. Long-term emotions (i.e. experienced at the time of the interview), composite score of 10 possible emotions: angry, fear, calm, anxious, shocked, depressed, numb, guilty, sad, and insecure 3. Intrusion symptom level, composite score | 1. Immediate emotions:  - bivariate analysis: of all risk/protective factores, only age group, monetary value of loss, sentimental value of loss, degree of disarrangement, and degree of territorial intrusion were associated with experiencing immediate emotions;   - victims between 40 and 59 old reported more immediate emotions than those between 25 and 39 years old;   - victims who had experienced a loss with sentimental value reported more immediate emotions than those who had not;   - victims who had experienced a higher degree of disarrangement reported more immediate symptoms than those who had experienced lower degrees;   - victims who had experienced a higher degree of territorial intrusion reported more immediate symptoms than those who had experienced lower degrees - multivariate analysis: not performed - path analysis: not performed  1. Long-term emotions:  - bivariate analysis: of all risk/protective factores, only sentimental value of loss and degree of disarrangement were positively associated with experiencing long-term emotions;   - victims who had experienced a loss with sentimental value reported more immediate emotions than those who had not;   - victims who had experienced a higher degree of disarrangement reported more immediate symptoms than those who had experienced lower degrees - multivariate analysis: not performed - path analysis: not performed  1. Intrusion symptom level:  - bivariate analysis: of all risk/protective factores, only monetary value of loss, sentimental value of loss, degree of disarrangement, and police handling of burglary were associated with suffering from intrusion symptoms;   - victims whose losses represented more monetary value reported higher levels of intrusion symptoms;   - victims who had experienced a higher degree of disarrangement reported higher levels of intrusion symptoms than those who had experienced lower degrees’;   - victims whose burglary had been handled through a house visit reported higher levels of intrusion symptoms than those whose burglary had been handled through telephone contact - multivariate analysis: not performed - path analysis: not performed |

1. Bivariate correlations of PTSD symptom levels with personality traits and coping strategies were not reported. [↑](#footnote-ref-1)
2. Bivariate correlations of mental health problems levels with personality traits and coping strategies were not reported. [↑](#footnote-ref-2)
3. Results for the other types of emotions were not reported. [↑](#footnote-ref-3)
4. Ibid. footnote 3. [↑](#footnote-ref-4)
5. Ibid. footnote 3. [↑](#footnote-ref-5)
6. Bivariate correlations were not reported for the other risk/protective factors and neither for the correlation of male sex with any of the other types of emotions. [↑](#footnote-ref-6)
7. Ibid. footnote 6. [↑](#footnote-ref-7)
